# Rare but relevant: Genetic liver disease in the general medical setting

**DOI:** 10.1016/j.clinme.2025.100535

**Published:** 2025-11-19

**Authors:** Sammi Allouni, Aftab Ala

**Affiliations:** a Synnovis Liver Molecular Genetics Laboratory, King’s College Hospital NHS Foundation Trust, London, UK; b Institute of Liver Studies, King’s College Hospital NHS Foundation Trust, London, UK

**Keywords:** Genetic liver disease, Wilson disease, Hereditary haemochromatosis, Alpha-1 antitrypsin deficiency, Genomic medicine

## Abstract

Genetic liver diseases are individually rare but collectively significant causes of chronic liver dysfunction in adults. Conditions such as Wilson disease, hereditary haemochromatosis and alpha-1 antitrypsin deficiency often present with vague or non-specific features, including fatigue, abnormal liver enzymes or extrahepatic manifestations. These features are easily misattributed to more common hepatic or systemic conditions, particularly in acute or general medical settings. Early recognition and investigation are crucial, as targeted treatments can prevent progression to end-stage liver disease, and timely referral enables cascade testing for at-risk relatives. With increasing access to genomic testing through systems such as the NHS Genomic Medicine Service in England, generalists play a key role in integrating genomics into routine care. This article provides a practical update on recognising, investigating and managing rare genetic liver conditions, aiming to support earlier diagnosis, better patient outcomes, and improved use of genomic services in frontline practice.

## Highlights

• Genetic liver diseases are often missed due to non-specific symptoms.
• Wilson disease, hereditary hemochromatosis and A1AT deficiency can present to generalists.
• Genomic testing enables earlier diagnosis and tailored treatment.
• Earlier recognition improves outcomes and supports family cascade testing.
• Generalists play a key role in integrating genomics into liver disease care.

## Graphical abstract

**Figure:**
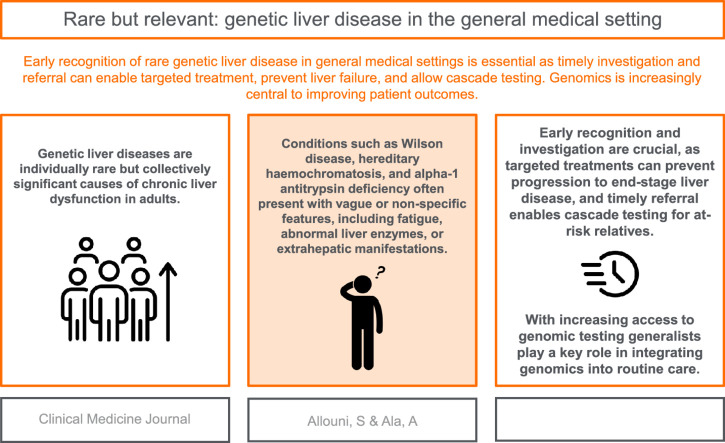

## Introduction

Rare liver diseases, although individually uncommon, collectively represent an important burden in adult general medicine. Many have an established genetic basis, but present non-specifically, often leading to misdiagnosis. Generalist physicians are vital for early recognition and referral. Timely diagnosis can prevent progression to end-stage liver disease, enable targeted treatment and support cascade testing of at-risk relatives.

Conditions like Wilson disease (WD), hereditary haemochromatosis (HC) and alpha-1 antitrypsin deficiency (A1ATD) are particularly relevant to adult practice. While diagnosis can be challenging, expanded access to genetic testing offers new opportunities to clarify causes and personalise care.

In England, genomic testing is delivered through the NHS Genomic Medicine Service (GMS), coordinated via seven regional genomic laboratory hubs. Equivalent services are commissioned separately in Scotland, Wales and Northern Ireland.

This article offers a guide to recognising, investigating and referring suspected genetic liver disease. Understanding these conditions supports earlier diagnosis and improved long-term outcomes, and helps embed genomic medicine into routine practice.

### Overview of key genetic liver conditions

Several genetic liver diseases are particularly relevant to generalist physicians. Despite differing pathophysiology, they often present with overlapping and non-specific features such as fatigue, abnormal liver enzymes or systemic symptoms. A high index of suspicion is essential, especially when liver dysfunction is unexplained or accompanied by extrahepatic features. This section highlights three well-established genetic liver conditions and briefly reviews some rarer but increasingly recognised disorders.

Wilson disease (WD) is a rare autosomal recessive disorder of copper metabolism caused by damaging changes in both copies of the *ATP7B* gene, which encodes a copper-transporting P-type ATPase. Impaired biliary copper excretion leads to hepatic copper accumulation. Presentation varies but often begins in adolescence or early adulthood. Adults may present with neuropsychiatric symptoms, abnormal liver function tests or acute hepatic failure.^1^

Diagnosis is frequently delayed due to its broad differential.^2^^,^^3^ Suggestive features include low serum ceruloplasmin, elevated 24-h urinary copper, and Kayser–Fleischer (KF) rings on slit-lamp examination. The Leipzig criteria (Table 1) aid assessment, with a score of ≥4 indicating probable diagnosis.^4^

**Table 1:** The Leipzig criteria for the scoring of Wilson disease.^6^

Typical clinical symptoms and signs	Score
KF rings	
Present	2
Absent	0
Neurological symptoms (eg mood disturbance, tremor, dystonia)^a^	
Severe	2
Mild	1
Absent	0
Serum ceruloplasmin	
Normal (>0.2 g/L)	0
0.1–0.2 g/L	1
<0.1 g/L	2
Coombs-negative haemolytic anaemia	
Present	1
Absent	0
Other tests	
Liver copper (in the absence of cholestasis)	
>5x ULN (>4 µmol/g)	2
0.8–4 µmol/g	1
Normal (<0.8 µmol/g)	−1
Rhodanine-positive granules^b^	1
Urinary copper (in the absence of acute hepatitis)	
Normal	0
1–2x ULN	1
>2x ULN	2
Normal, but >5x ULN after D-penicillamine	2
*ATP7B* mutation screening – clinical indication ID R172 on NHSE Genomics Test Directory (single gene sequencing for *ATP7B)*. *Note that the R171 gene panel is available for the identification of some conditions that may mimic Wilson disease (*eg *PFIC3). The R98 large gene panel is available for the identification of rare variants in other copper metabolism genes.*	
On both chromosomes detected	4
On 1 chromosome detected	1
No mutations detected	0
Total score	Evaluation:
4 or more	Diagnosis established
3	Diagnosis possible, more tests needed
2 or less	Diagnosis very unlikely

^b^If no quantitative liver copper available, ^a^or typical abnormalities at brain magnetic resonance imaging.

KF, Kayser–Fleischer; ULN, Upper limit of normal.

Genetic testing typically involves sequencing the coding region of *ATP7B* and can support or confirm diagnosis. If results are inconclusive, broader panel testing or exclusion of mimics (eg PFIC3) may be helpful. As testing is not usually confined to specific variants, results may include variants of uncertain significance (VUS). Chelation therapy can be lifesaving and improve both hepatic and neurological outcomes.^5^

Hereditary haemochromatosis (HC) is an autosomal recessive disorder of incomplete penetrance, meaning that not all individuals with disease-associated genotypes will develop clinical symptoms. The *HFE* gene encodes a transmembrane protein that regulates iron absorption via interaction with transferrin and its receptor (TfR). When both copies of the gene are affected, excessive intestinal iron uptake can occur, potentially leading to progressive iron overload. However, clinical expression varies by age, sex, and other genetic and environmental factors.

While classically associated with cirrhosis, diabetes and skin hyperpigmentation, most patients present with subtler signs such as fatigue, mildly elevated liver enzymes, arthropathy, or a family history of iron-related disease.^7^^,^^8^ Iron studies typically show elevated serum ferritin and transferrin saturation of ≥45%.

Most cases result from homozygosity for the C282Y variant in *HFE*. Targeted genetic testing for common *HFE* mutations is widely available, including via primary care (R95 on the National Genomics Test Directory). This is usually sufficient to confirm diagnosis. However, routine assays do not sequence the full *HFE* gene, and broader panels may be needed to detect ultra-rare causes.

Early diagnosis is key, as regular phlebotomy is an effective treatment that can prevent complications.^9^ Table 2 outlines key diagnostic criteria.

**Table 2:** Basic diagnostic criteria for hereditary haemochromatosis (HC).

Typical clinical symptoms and signs	Comments
Transferrin saturation (TS)	
≥50% in men; ≥45% in women	Most sensitive early indicator of iron overload
Serum ferritin (SF)	
≥300 µg/L in men; ≥200 µg/L in women	Elevated with iron overload, but may also be raised in inflammation
Repeat TS and SF	
Persistently increased, excluding secondary causes	Suggests provisional primary iron overload
Targeted *HFE* genotyping – clinical indication ID R95 on NHSE Genomics Test Directory – Targeted genotype testing for C282Y and H63D only. *Note that the R96 gene panel is available for the detection of rare variants in HFE and other iron metabolism genes.*	
C282Y homozygosity C282Y/H63D compound heterozygosity	Associated with significant genetic predisposition to iron overload Associated with small genetic predisposition to iron overload
Clinical features	
Fatigue, arthropathy (esp. 2nd/3rd MCPs), ↑LFTs	Subtle presentations common; classic triad (cirrhosis, diabetes, skin pigmentation) is rare
Exclusion of secondary causes	
Alcohol, liver disease, metabolic syndrome, etc.	Important to exclude other causes of hyperferritinaemia before diagnosis
Treatment response	
Reduction in TS and SF with phlebotomy	Both diagnostic and therapeutic; may support clinical diagnosis

LFTs, Liver function tests; MCPs, Metacarpophalangeal.

Alpha-1 antitrypsin deficiency (A1ATD) is traditionally described as an autosomal co-dominant condition, meaning that both copies of the *SERPINA1* gene influence clinical presentation. It also shows incomplete penetrance. The translated protein (alpha-1 antitrypsin; A1AT) is a serine protease inhibitor that protects tissue by neutralising neutrophil elastase, an enzyme capable of degrading structural proteins during inflammation.

In the context of A1ATD, it is commonplace to use nomenclature related to the protein phenotype observed when separating variant A1AT proteins using isoelectric focusing (phenotyping). Pi*M denotes normal (wild type) A1AT, while Pi*Z and Pi*S are the most common mutant isoforms. This is distinct from the underlying *SERPINA1* variant nomenclature obtained by genotyping (eg E342K for the Z allele).

A1ATD primarily affects the lungs and liver. While panacinar emphysema in younger smokers is a classic feature, liver disease ranging from asymptomatic transaminitis to cirrhosis or primary hepatocellular carcinoma can be the initial or sole manifestation.^10^

Lung and liver injury arise via distinct mechanisms. In the lung, reduced A1AT levels allow unopposed neutrophil elastase activity. In the liver, misfolded Z-A1AT protein polymerises and accumulates in hepatocytes, causing toxic gain-of-function injury. Alleles like Z affect both organs, while null alleles (that cause loss of function without polymer formation) typically spare the liver.

Homozygosity for the Z allele (Pi*ZZ) carries the highest risk, but even individuals with a single variant copy (heterozygotes) show an increased liver disease burden, with higher levels of fibrosis being reported in those of Pi*SZ genotype as compared with Pi*MZ.^11^

A1ATD should be considered in adults with unexplained liver disease, especially with early-onset pulmonary symptoms or relevant family history. Low serum A1AT levels raise suspicion, and the diagnosis is confirmed by phenotyping or genotyping.^12^

While current management of the hepatic manifestations of A1ATD is largely supportive, major therapeutic advances are anticipated in the coming years. Because lung and liver injury arise from distinct mechanisms, treatments targeting one may not benefit, and may even exacerbate, the other. Liver-directed therapies aim to stabilise or improve folding and secretion of the variant Z-A1AT protein (eg chaperone-based therapies), or reduce its production using small interfering RNA (siRNA) that degrades variant *SERPINA1* messenger RNA (mRNA; silencing therapy). Both approaches show promise in reducing liver injury.^13^^,^^14^ Emerging trials now focus on correcting *SERPINA1* variants at the DNA or RNA level to restore functional A1AT, offering a potentially disease-modifying strategy that could address both hepatic and pulmonary manifestations. Key diagnostic criteria are summarised in Table 3.

**Table 3:** Basic diagnostic criteria for alpha-1 antitrypsin deficiency (A1ATD).

Typical clinical symptoms and signs	Comments
Serum A1AT level	
Low (usually ≤1.2 g/L; reference range varies by lab).	Suggests deficiency, but can also be elevated in inflammation (acute-phase reactant). It is important to ensure that thresholds used are sufficiently sensitive to identify Pi*MZ individuals
Phenotyping	
Z allele most clinically relevant for liver disease. Pi*ZZ = High risk Pi*SZ = Intermediate risk Pi*MZ = Intermediate risk	Isoelectric focusing separates specific A1AT protein variants
Targeted *SERPINA1* genotyping – clinical indication ID R191 on NHSE Genomics Test Directory (targeted genotype testing for E264V and E342K only). *Note that identifying rare variants requires sequencing of SERPINA1, which is included in the R171 gene panel.*	
E342K (Z allele) most clinically relevant for liver disease. E342K/E342K (Pi*ZZ) = high risk E264V/E342K (Pi*SZ) = intermediate risk E264V/E342K (Pi*SZ) = intermediate risk	Targeted genotyping identifies specific known pathogenic alleles (eg Z, S, null)
Clinical features	
Unexplained LFTs; cirrhosis; early-onset emphysema	May be hepatic or pulmonary; consider in adults with both or family history
Liver biopsy (rarely needed)	
PAS-positive, diastase-resistant globules in hepatocytes Z-A1AT immunohistochemistry	Seen in severe deficiency; not routinely required for diagnosis. Stain specific to the Z-polymer form of the protein. Can demonstrate intracytoplasmic accumulation of polymerised Z-A1AT within hepatocytes
Investigation of other causes	
Alcohol, viral hepatitis, MASLD	Important in interpreting abnormal LFTs

LFTs, Liver function tests; PAS, Periodic Acid–Schiff; MASLD, Metabolic dysfunction-associated steatotic liver disease.

Other inherited liver disorders may also present to generalists, albeit more rarely. As with previously discussed conditions, their wide spectrum of severity and often non-specific features increases the risk of misdiagnosis.

Progressive familial intrahepatic cholestasis (PFIC) involves bile acid transport defects and may present with pruritus and cholestasis.^15^ Though traditionally considered autosomal recessive, emerging evidence suggests that even heterozygous variants can contribute to disease.^16^ Notably, heterozygous variants in *ABCB4* and *ABCB11* are known to predispose to intrahepatic cholestasis of pregnancy (ICP), indicating that disease severity correlates with residual transporter function rather than following simple Mendelian inheritance.^17^

Disorders arising from ductal plate malformation, such as congenital hepatic fibrosis and Caroli disease, result from abnormal embryonic bile duct development and may present with portal hypertension, cholestasis or recurrent cholangitis. These conditions often co-occur with renal cystic disease, so liver abnormalities alongside kidney involvement should raise clinical suspicion.

Alagille syndrome is an autosomal dominant condition typically involving cholestasis, cardiac anomalies and dysmorphic features. Expressivity varies widely, even within families, and milder adult forms may be overlooked.^18^

Finally, hepatic presentations can also occur in mitochondrial disorders and various inherited metabolic conditions, both increasingly recognised due to broader gene panel testing.^19^^,^^20^

Table 4 summarises key features and diagnostic considerations of these disorders.

**Table 4:** Key features and diagnostic considerations in other relevant inherited liver disorders.

Condition	Typical features	Diagnostic clues and tools
PFIC (types 1–3 and related)	Cholestasis, pruritus Normal/low GGT in PFIC1&2 High GGT in PFIC3	Cholestatic LFTs without obstruction; gene panel findings eg *ATP8B1* (PFIC1), *ABCB11* (PFIC2), *ABCB4* (PFIC3). Relevant genes can be tested using *clinical indication ID R171 on NHSE Genomics Test Directory*
Intrahepatic cholestasis of pregnancy (ICP)	Pruritis in pregnancy, raised bile acids; symptoms resolve postpartum	Consider screening for *ABCB4* or *ABCB11* variants if recurrent, severe or family history present, bile acids guide obstetric management. Relevant genes can be tested using *clinical indication ID R171 on NHSE Genomics Test Directory*
Ductal plate malformation (eg Caroli disease)	Portal hypertension, cholestasis, recurrent cholangitis	Characteristic biliary tree changes on imaging (eg segmental/saccular duct dilation); consider when liver disease coexists with renal cystic abnormalities, liver histology may demonstrate ductal plate malformation. Relevant genes can be tested using *clinical indication ID R173 on NHSE Genomics Test Directory*
Alagille syndrome	Cholestasis, cardiac defects, facial features, butterfly vertebrae	Suggestive family history; bile duct paucity on liver biopsy; genetic findings (*JAG1* or *NOTCH2*). Relevant genes can be tested using *clinical indication ID R171 on NHSE Genomics Test Directory*
Mitochondrial hepatopathies	Multisystem involvement (eg neuromuscular symptoms)	Elevated lactate; genetic findings (mtDNA and nuclear mitochondrial genes); neuroimaging; muscle/liver biopsy. Relevant genes can be tested using *clinical indication ID R317 on NHSE Genomics Test Directory (nuclear mitochondrial genes only)*
Inherited metabolic disorders	Hypoglycaemia, failure to thrive, neurological signs, hepatomegaly	Elevated metabolites on plasma/urine screening; relevant gene panel findings (eg urea cycle genes, glycogen storage genes). Relevant genes can be tested using *clinical indication ID R98 on NHSE Genomics Test Directory*

PFIC, Progressive familial intrahepatic cholestasis; LFTs, Liver function tests; GGT, Gamma-glutamyl transferase; mtDNA, Mitochondrial DNA.

### Role of genomics in diagnosis

Genomic testing is increasingly central to diagnosing rare liver disease. For generalists, recognising when to suspect a genetic cause and knowing how to access testing is essential to early diagnosis and referral. In HC and A1ATD, targeted genotyping of *HFE* and *SERPINA1* variants is accessible in primary care and usually sufficient. Full *ATP7B* sequencing is usually required to confirm WD, however, as causative mutations are diverse and often private.

For patients with unexplained liver dysfunction, early-onset cholestasis or syndromic features, referral to hepatology or clinical genetics may lead to broader testing through NHS GMS panels. As access expands, variants of uncertain significance are increasingly encountered, often requiring multidisciplinary interpretation. Generalists should initiate testing where appropriate and refer complex cases for specialist input.

### Implications for management and referral

Management depends on the underlying condition. In HC, early venesection is effective. WD is treated with chelators and/or zinc to reduce copper burden. No disease-modifying therapy is currently available for the hepatic manifestations of A1ATD, but monitoring and lifestyle modification are essential, and significant genetic therapies are emerging. Lung disease may benefit from intravenous A1AT augmentation therapy.

Referral to hepatology or clinical genetics is appropriate when there is unexplained liver dysfunction, relevant family history or extrahepatic features. Cascade testing may identify at-risk relatives. Where cirrhosis or liver failure develops, early referral for transplant assessment is essential.

## Conclusion

Genetic liver diseases are rare but clinically important, and early recognition can significantly alter their clinical course. Presentations are often non-specific and easily misattributed, especially in busy general medical settings. Generalists are frequently the first to encounter these patients and play a vital role in raising suspicion, initiating appropriate investigations and facilitating timely referral. With expanding access to genomic testing and emerging targeted therapies, early diagnosis can improve long-term outcomes and enable cascade testing to benefit affected families.

## CRediT authorship contribution statement

**Sammi Allouni:** Writing – original draft. **Aftab Ala:** Writing – review & editing, Conceptualization.

## Declaration of competing interest

Aftab Ala is the associate editor of ClinMed. If there are other authors, they declare that they have no known competing financial interests or personal relationships that could have appeared to influence the work reported in this paper.
